# Urine neutrophil gelatinase-associated lipocalin and urine output as predictors of the successful discontinuation of continuous renal replacement therapy in critically ill patients with acute kidney injury

**DOI:** 10.1186/s12882-020-02035-w

**Published:** 2020-08-28

**Authors:** Josefine Thomsen, Ulrik Sprogøe, Palle Toft

**Affiliations:** 1 Department of Anaesthesiology and Intensive Care, Odense University Hospital, Odense, Denmark; 2 Department of Clinical Immunology, Odense University Hospital, Odense, Denmark

**Keywords:** Renal replacement therapy, Critical care, Acute kidney injury, Intensive care unit, Dialysis, Urine neutrophil gelatinase-associated lipocalin

## Abstract

**Background:**

Continuous renal replacement therapy (CCRT) is a frequently used modality for the support of intensive care patients with acute kidney injury (AKI). Nevertheless, there are no objective criteria for the discontinuation of CRRT. The purpose of this study was to investigate whether urine neutrophil gelatinase-associated lipocalin (uNGAL) alone or in combination with urine output could be used as a diagnostic test for renal function recovery in ICU patients on CRRT.

**Methods:**

This was a single-centre prospective observational study including patients with acute kidney failure needing CRRT. Sixty-nine patients were enrolled, and 54 completed the study. Of the 54 patients, 22 recovered renal function (REC), defined as dialysis independency at 72 h from discontinuation, while 32 patients did not (NREC). Urine NGAL was measured at 0, 6, 12, and 24 h after CRRT discontinuation. The cumulated urine output was measured for 24 h prior to discontinuation and at 6, 12, and 24 h after discontinuation. Missing uNGAL values were calculated by interpolation. The Youden’s index was used to calculate cut-off values in order to define uNGAL and urine output single variable and 2-variable diagnostic tests with the optimum prediction of successful CRRT discontinuation.

**Results:**

Baseline characteristics at CRRT initiation were similar between groups. Compared to the NREC group, the REC group had significantly higher urine output (*p* < 0.0001) and lower uNGAL (*p* < 0.001) at all time points, except for uNGAL at 24 h (*p* < 0.24). The best uNGAL predictor for successful CRRT discontinuation was uNGAL at 6 h after discontinuation (predictive value 80%). The best single predictor was cumulated urine output 24 h before discontinuation (predictive value 85%). The combinations of uNGAL at 6 h (cut-off 1650 μg/L) with cumulated urine output 24 h prior to discontinuation (cut-off 210 ml) proved to be the superior tests (using either “or” or “and”), with predictive values of 93% (successful CRRT discontinuation) and 92% (dialysis dependency).

**Conclusions:**

With a predictive value of 93%, the combination of uNGAL at 6 h after and the cumulated urine output 24 h prior to CRRT cessation proved to be the best diagnostic test for successful CRRT discontinuation in ICU patients.

**Clinical trial registration:**

N/A

## Background

Acute kidney injury (AKI) is a common complication in patients admitted to an intensive care unit (ICU). An international multicentre study found that 13.5% of patients admitted to the ICU required renal replacement therapy (RRT) [1]. The preferred choice of dialysis modality is continuous renal replacement therapy (CRRT), thereby obtaining better solute control and more stable haemodynamics. However, despite the frequent use of CRRT, there is no consensus regarding its discontinuation. Ideally, the physician would have access to a test with high diagnostic sensitivity for predicting renal recovery. This is, however, complicated by the complex physiology and individualized presentation of the critically ill patient. In clinical practice, CRRT is discontinued on an individual basis, i.e., when the physician assesses that the general state of the patient could indicate renal recovery. Urine output is currently the most valid predictor for successful discontinuation [2–6]. Other less predictive variables are serum and urine creatinine, Sequential Organ Failure Assessment (SOFA) score, dialysis time, age, co-morbidities, and 2-h creatinine clearance [2–7]. In recent years, specific biomarkers have been associated with renal injury and renal recovery. Currently, the most promising and thoroughly explored biomarker is neutrophil gelatinase-associated lipocalin (NGAL). NGAL has already been shown to be a very early predictor of AKI [8–13]. In cases in which the duration of trauma to the kidney is known, such as following organ transplantation and cardiac surgery, NGAL has shown excellent results. The landmark trial by Mishra and colleagues [11] from 2005 showed that urine NGAL (uNGAL) 2 h after cardiopulmonary bypass had an area under the receiver operating curve (ROC) of 99.8% for the prediction of AKI. In the same study, the serum creatinine increased with a 1–3 day delay. Urine NGAL is an expression of ongoing *damage* to the kidney, whereas creatinine reflects the *function* of the kidney. A decrease in uNGAL might therefore represent renal recovery. Few studies have focused on the ability of uNGAL to predict renal recovery in critically ill patients at the time of discontinuation of dialysis [14–16]. In these studies, uNGAL was determined 24 h or later after discontinuation. It may be argued that the longer the timeframe from discontinuation until the uNGAL level is determined, the less clinically relevant the biomarker becomes.

The objectives of the present study were to investigate whether uNGAL as a diagnostic test in the interval 0–24 h after discontinuation of CRRT is able to predict renal recovery in critically ill patients, and whether a diagnostic test combining uNGAL with urine output could be an even better predictor of renal recovery.

## Methods

### Setting

This single-centre prospective observational study was conducted in the tertiary ICU at Odense University Hospital between May 2016 and April 2018. Patients with AKI, according to the RIFLE criteria, who were admitted to the ICU were included at the start of CRRT. The exclusion criteria were age < 18 years, chronic kidney failure and missing consent. Patient follow-up was 3 months after discharge from the ICU. The decision as to when to initiate/discontinue CRRT was at the discretion the ICU physicians on duty, and it was based on the complex physiology and individualized presentation of the critically ill patient/critically ill patient in recovery.

Continuous veno-venous haemodialysis (CVVHD) was the preferred renal replacement modality with multi-filtrate CiCa dialysate (Fresenius Medical Care, Bad Homburg, Germany). If the patient was otherwise ready to be transferred to a regular clinical ward, haemodialysis (HD) was initiated instead of CRRT, i.e., HD was usually initiated at the clinical ward or shortly before transfer out of the ICU.

### Endpoint

The successful endpoint with regard to the diagnostic test of uNGAL and urine output was defined as 3 dialysis-free days (72 h) following the discontinuation of CRRT.

### Data collection

Demographic and basic clinical information together with a baseline urine sample for uNGAL analysis were obtained at study inclusion. Serum creatinine (S-Cr) and CRP at inclusion (Table 1) refer to samples from 06.00 a.m. of the calendar day of CRRT initiation. For patients who were admitted later than 06:00 a.m. and initiated CRRT the same day, S-Cr and CRP at the time of ICU admission were used.

**Table 1:** Baseline characteristics at continuous renal replacement therapy initiation

Clinical variables	Recovery (*n* = 22)	Non-recovery; CRRT re-initiation (*n* = 20)	Non-recovery; Haemodialysis initiation (*n* = 12)	*P*-value
Male/female	16/6 (73%/27%)	11/9 (55%/45%)	9/3 (75%/25%)	0.56
Age, years	77 [42–83]	70 [51–81]	68 [30–83]	0.84
BMI, kg/m^2^	26 [19–35]	27 [19–38]	25 [24–37]	0.45
SOFA, highest value	13 [7–18]	15 [11–19]	13 [8–21]	0,20
APACHE II	27 [18–40]	29 [21–43]	26 [18–38]	0.71
SAPS II	56 [30–75]	65 [44–86]	58 [38–78]	0.10
Hypertension	9 (41%)	11 (55%)	7 (58%)	0.76
Malignity	6 (27%)	10 (50%)	2 (17%)	0.56
CVVH/CVVHD	0/22 (0/100%)	4/16 (20/80%)	0/12 (0/100%)	0.14
RIFLE-F	21 (95%)^a^	20 (100%)	12 (100%)	0.41
Furosemide, mg/day	0 [0–554]	80 [0–534]	100 [0–480]	0.44^d^
Mean arterial pressure, mmHg	69 [62–85]	70 [63–93]	78 [59–113]	0.23
Creatinine, μmol/L	186 [84–621]	194 [87–531]	238 [90–765]	0.99
Creatinine clearance, ml/min	19 [7–45]	15 [8–27]	17 [5–26]	0.11
C-Reactive Protein (CRP), mg/L	216 [7–454]	139 [13–266]	108 [34–423]	0.18
Urine output, ml/hour ^b^	18 [0–50]	8 [0–97]	0 [0–52]	0.40^d^
Urine output, ml/day ^c^	398 [103–1488]	326 [8–1750]	120 [13–2550]	0.23^d^
Noradrenaline, μg/kg/min	0.35 [0.09–1.26]	0.28 [0.07–1.32]	0.12 [0.02–1.10]	0.35^d^
Nephrotoxic medicine including contrast.	8 (36%)	12 (60%)	6 (50%)	0.18
Presumed primary cause of AKI:				0.56
Prerenal	8 (36%)	5 (25%)	2 (17%)	
Sepsis	12 (55%)	13 (65%)	4 (33%)	
Glomerulonephritis	0	0	1 (8%)	
Rhabdomyolysis	1 (4,5%)	1 (5%)	3 (25%)	
Microthrombosis/vasculitis	1 (4,5%)	1 (5%)	2 (17%)	
Primary reason for ICU admission:				0.62
Septic shock/sepsis	9 (41%)	103 (65%)	6 (51%)	
Trauma	1 (5%)	2 (10%)	0	
AKI	5 (23%)	1 (5%)	2 (17%)	
Respiratory failure	4 (18%)	2 (10%)	3 (34%)	
Low cardiac output	3 (14%)	2 (10%)	1 (8%)	
Urine-NGAL, μg/L	2645 [279–27,543]	2894 [749–18,537]	3619 [128–30,170]	0.57^d^
Time, ICU admission to CRRT initiation/study inclusion, days	1 [0–7.4]	2 [0–5.9]	1 [0–12.1]	0.94

*P-value was calculated between the recovery and non-recovery (CRRT-re-initiation and haemodialysis) groups. CRRT, continuous renal replacement therapy; SAPS II, Simplified Acute Physiology Score II; SOFA, Sequential Organ Failure Assessment; APACHE II, Acute Physiology and Chronic Health Evaluation II; CVVH, continuous veno-venous haemofiltration; CVVHD, continuous veno-venous haemodialysis; AKI, acute kidney injury; NGAL, neutrophil gelatinase-associated lipocalin. Categorical variables are expressed as numbers (percentages). Continuous variables are expressed as medians [10–90% quantiles]*

*a) RIFLE-I was recorded for one patient. b) Mean urine output 6 h before dialysis initiation; if urine output < 5 ml in one hour, then it was recorded as 0 ml. c) Urine output 24 h before CRRT initiation. d) Log10 transformation was used to calculate the p-value*

At the time of the first cessation of CRRT, the following physiological and laboratory variables were collected: uNGAL at the time of cessation (0 h) and at 6, 12, and 24 h (uNGAL 0 h, uNGAL 6 h, uNGAL 12 h, uNGAl 24 h) thereafter; mean arterial pressure at 0, 6, 12 and 24 h; use of diuretics at 6, 12, and 24 h (note that in our ICU unit, diuretics are not given during CRRT treatment); s-Cr, creatinine clearance, and CRP at 0 and 24 h; cumulated urine output for 24 h prior to CRRT cessation (UOC24pre) and 24 h after CRRT cessation (UOC24post); average hourly urine output during the first 6 h (UOA6) and during the first 12 h (UOA12) after cessation; and the time from CRRT cessation until the re-initiation of CRRT.

### Outcome data

The outcomes were dialysis dependence and mortality at 3 months after ICU discharge.

### Urine NGAL collection and analysis

Urine specimens for analysing uNGAL were collected primarily from the urinary catheter and, if that was not possible, from the urinary catheter bag. The urine sample was immediately transferred to a − 80 °C freezer.

Urine NGAL was measured using an automated, turbidimetric immunoassay (NGAL Test Reagent Kit, BioPorto Diagnostics, Denmark) on a Roche cobas 8000 analyser (Roche Diagnostics, Rotkreutz, Switzerland). The 95% reference range for uNGAL (4.3–204 μg/L) was based on uNGAL measurements in 50 healthy adults (laboratory staff and their family members, 25 males and 25 females) aged 21–66 years using the same uNGAL method as employed in the study.

### Data processing and analysis

Study data were managed using REDCap (REDCap Consortium, Vanderbilt University Medical Center, Nashville, TN) electronic data capture tools in the Odense Patient Data Explorative Network (OPEN), hosted by the University of Southern Denmark and Odense University Hospital. All data were transferred to Excel 16 (Microsoft, Redmond, WA) for Mac and analysed using JMP 14.1 (SAS Institute, Cary, North Carolina) statistical software.

To validate the diagnostic tests for the successful discontinuation of CRRT, the patients were divided into the “recovery” (REC) and “non-recovery, all” (NREC-A) groups. Successful recovery was defined as the patient being independent from dialysis for at least 72 h. The NREC-A group was subdivided according to whether the patient initiated haemodialysis (NREC-H) or reinitiated CRRT (NREC-C). The patients were excluded from analysis if they had died before discontinuation or if active treatment of the patient’s basic condition had been discontinued while they were on CRRT. Each patient was included only once.

Demographic variables and variables at discontinuation were compared between the REC and NREC-A groups using Student’s t-tests and Wilcoxon non-parametric tests for continuous variables. Fisher’s exact tests were used for categorical variables. *p* < 0.05 was considered statistically significant.

The NGAL values were shown to be log-normally distributed. Linear regression analysis of the correlations between log uNGAL at 6 h and log uNGAL at 12 h and between log uNGAL 6 at hours and log uNGAL at 0 h was used to calculate the missing uNGAL 6 h values or missing uNGAL 0 h values by interpolation.

For all uNGAL (at 0, 6, 12 and 24 h) and urine output (UOC24pre, UOC24post, OUA6 and OUA12) measures, the area under the receiver operating characteristic (ROC) curve, sensitivity, specificity, the negative predictive value (NPV), and the positive (“positive” outcome defined as the patient being dialysis dependent) predictive value (PPV) were calculated using Excel 16 (Microsoft, Redmond, WA) and Prism 8 (GraphPad, San Diego, CA). Youden’s indices (defined as sensitivity + specificity - 1,00) were calculated to determine the optimum cut-off values, i.e. highest combined sensitivity and specificity. Each of the identified uNGAL and urine output optimum cut-off values were permutated in “and” and “or” expressions to identify the combination of the 2 variables yielding the highest sensitivity or NPV [17], i.e., a “test” based on uNGAL and urine output, if negative, giving the best prediction of renal function recovery, i.e., successful CRRT discontinuation.

To estimate the increase in diagnostic performance of 2-variable models in comparison with the relevant single variable models, net classification index (NRI) was calculated as (sensitivity + specificity [2-variable model]) - (sensitivity + specificity [single variable model]), with a positive value indicating higher diagnostic performance of the 2-variable model.

## Results

**Fig. 1:**
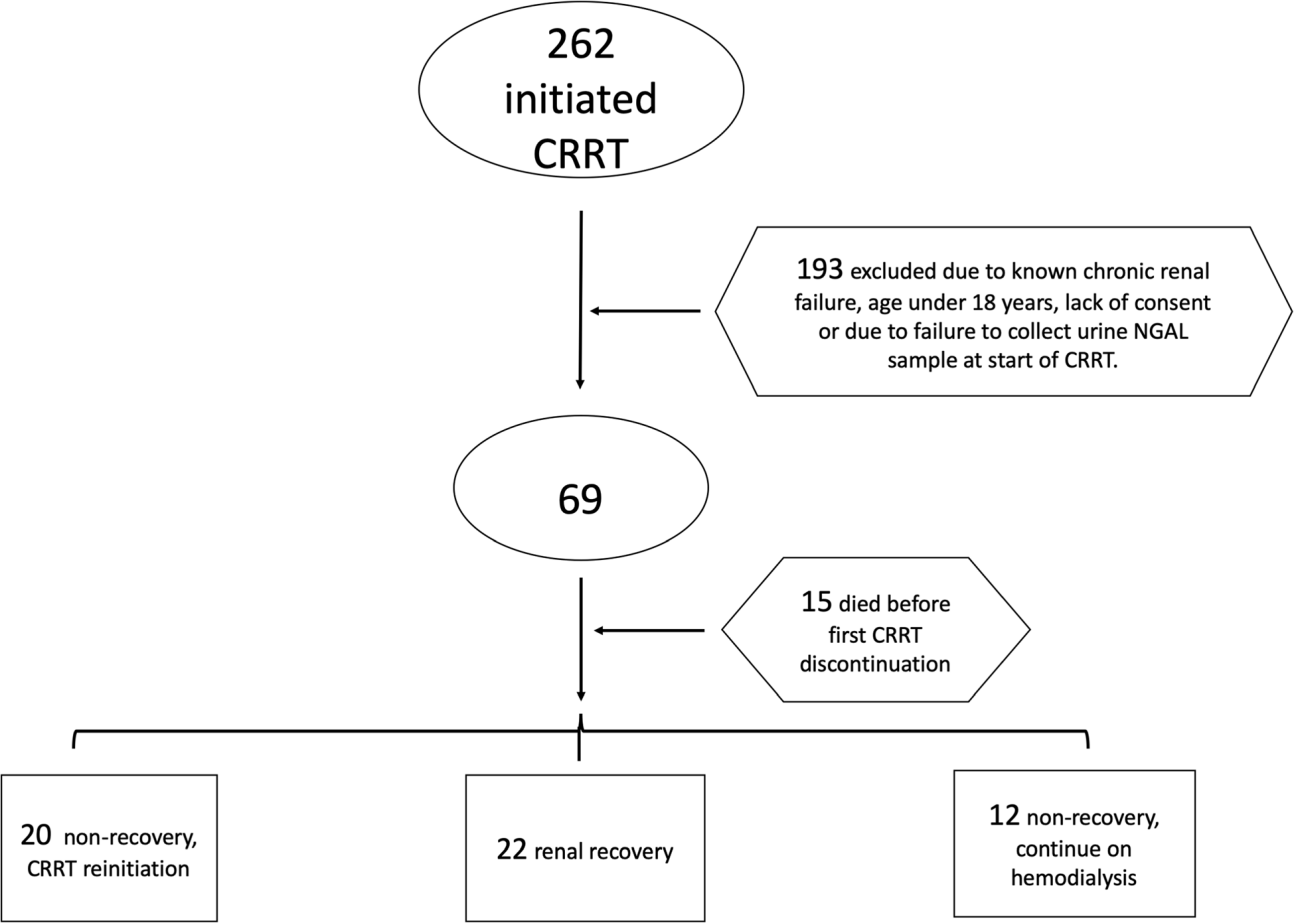
Flow chart of the inclusion of study subjects -no need for legend

Sixty-nine patients were enrolled in the study. A total of 262 patients were treated with CRRT at the Odense University Hospital during the 2-year study period. A total of 193 (74%) patients were excluded, either because they did not meet the inclusion criteria or because of failure to collect urine within the inclusion window. In addition, 15 out of the 69 (22%) patients died while on CRRT. Of the remaining 54 patients, 22 (41%) experienced renal function recovery (REC), whereas 32 (59%) patients did not recover renal function (NREC-A). This NREC-A group of 32 was further subdivided into the NREC-C (20 [63%] patients) and NREC-H (12 [37%] patients) subgroups (Fig. 1). One patient in the REC group was placed back on CRRT after 92 h of dialysis independence.

The baseline characteristics of the three groups, REC, NREC-H, and NREC-C, are summarized in Table 1. The median age, sex, and number of comorbidities were similar among the groups. Table 2 shows the variables at the time of and following the discontinuation of CRRT. The REC group had significantly lower uNGAL levels at 0, 6, and 12 h after CRRT discontinuation compared to the NREC-A group. The average UO6, UO12, UOC24post, and UOC24pre levels were significantly higher in the REC group than in the NREC-A. There was a tendency towards lower s-Cr and higher creatinine clearance at 24 h after CRRT discontinuation in the REC group compared to the NREC-A group. Number of days on CRRT before discontinuation was significantly lower in the REC group.

**Table 2:** Variables at the time of discontinuation of continuous renal replacement therapy

Variablep	n	Recovery (n = 22)	Non-recovery; CRRT re-initiation (n = 20)	Non-recovery; Haemodialysis initiation (n = 12)	p-value
Urine NGAL					
time 0, μg/L	42	1370 [154–8002]	8934 [2095–40,000]	8726 [360–35,449]	0.0003^d^
6 h, μg/L	37	465 [56–3978]	2966 [1985–29,011]	2854 [120–6055]	0.0006^d^
12 h, μg/L	39	436 [95–3780]	1976 [798–16,972]	2148 [54–3825]	0.0008^d^
24 h, μg/L	31	455 [100–3337]	2290 [874–17,665]	295 [32–1624]	0.24^d^
MAP					
time 0, mmHg	54	80 [67–103]	71 [62–94]	82 [67–96]	0.12
6 h, mmHg	52	78 [66–105]	73 [66–105]	78 [64–106]	0.30^d^
12 h, mmHg	50	75 [67–106]	73 [65–105]	80 [68–103]	0.42
24 h, mmHg	46	75 [67–106]	75 [67–103]	83 [68–113]	0.60
Furosemide					
6 h., mg	52	70 [0–240]	70 [0–246]	5 [0–240]	0.94
12 h., mg	50	95 [0–480]	95 [0–480]	65 [0–480]	0.66
24 h., mg	46	150 [0–853]	240 [15–936]	70 [0–936]	0.50
Use of other diuretics		4 (18%)	4 (20%)	6 (50%)	0.52
Urine output 24 h prior to CRRT (time 0) discontinuation, ml	54	500 [87–2140]	100 [31–533]	20 [0–575]	< 0.0001
Urine output after discontinuation					
6 h, ml/hr	52	65 [13–266]	8 [0–47]	3 [0–50]	< 0.0001
12 h, ml/hr	50	85 [27–197]	10 [0–60]	3 [0–54]	< 0.0001
24 h, ml	46	2340 [828–4488]	480 [0–2952]	240 [0–3828]	< 0.0001
Time to re-initiation of dialysis, hrs			24 [6–64]	48 [20–71]	
Time on CRRT, days	54	4 [2–10]	8 [4–20]	5 [2–18]	0,001^d^
Mechanical ventilation, days	54	4 [0–23]	14 [0–32]	2 [0–18]	0.18
ICU, days	54	10 [2–32]	17 [5–38]	9 [3–20]	0.22
Creatinine					
At discontinuation, μmol/L 24 h after	54	98 [51–250]	130 [67–289]	157 [82–314]	0.18
discontinuation, μmol/L	46	134 [67–340]	191 [119–409]	235 [135–433]	0.03
Creatinine clearance					
At discontinuation, ml/min 24 h after	54	62 [17–90]	43 [21–85]	37 [18–75]	0.08
discontinuation, ml/min	46	38 [16–82]	28 [15–52]	22 [12–41]	0.02
C-reactive protein (CRP)					
At discontinuation, mg/L 24 h after	54	135 [35–267]	89 [22–260]	80 [16–172]	0.09
discontinuation, mg/L	46	125 [26–238]	100 [20–320]	65 [13–152]	0.28

*P-value was calculated between the recovery and non-recovery (CRRT-re-initiation and haemodialysis) groups. d) Log10 transformation was used to calculate the p-value*

Notable differences between the NREC-H and NREC-C groups is that the NREC-H group displayed an 8-fold lower mean uNGAL 24 h, and also a 5-fold lower mean value of UOC24pre compared to that of the NREC-C group. However, none of these differences reached statistical significance.

A comparison of REC versus NREC-C and REC versus NREC-H can be found in the appendix.

Out of the 345 potential samples, 214 (62%) urine samples were obtained for NGAL analysis. Fifty-seven samples were lost because the patient died while on dialysis, 30 samples were not obtained due to anuria, 31 samples were not collected (forgotten), and 13 samples were missed because dialysis was reinitiated less than 24 h after discontinuation.

There was a good statistical correlation between log uNGAL 6 h and log uNGAL 0 h (r^2^ = 0.81) and between log uNGAL 6 h and log uNGAL 12 h (r^2^ = 0.79), indicating that missing uNGAL 6 h or uNGAL 0 h values could be interpolated using the aforementioned linear regression models. From interpolation based on the uNGAL 0 h values, 13 interpolated uNGAL 6 h values were obtained. Likewise, based on the uNGAL 12 h values, 1 interpolated uNGAL 6 h value was obtained. Finally, based on the uNGAL 6 h values, 8 interpolated uNGAL 0 h values were obtained. Thus, 51 uNGAL 6 h and 50 uNGAL 0 h values were available for further analysis. The 3 missing uNGAL 6 h values were associated with patients in the NREC-A group, all of whom had a UOC24pre < 100 mL.

The overall ICU mortality was 46% (29 patients). At 3 months after ICU discharge, the mortality rate increased to 55% (38 patients). Among the 31 patients who were alive 3 months after ICU discharge, only 2 required dialysis.

The area under the ROC curve (AUC), sensitivity and specificity, NPV, and PPV for the different urine outputs and uNGAL values for the prediction of the successful discontinuation of CRRT are presented in Table 3. For uNGAL 12 h and uNGAL 24 h, AUC based on the ROC-curves were 0.82 and 0.64, respectively. However, due to high fractions of missing samples – which were not accessible for interpolation – of uNGAL at these two time points (uNGAL 12 h: 15/54 samples and uNGAL 24 h 21/54 samples), uNGAL 12 h and uNGAL 24 h were not further explored as test variables.

**Table 3:** Diagnostic test parameters for urine neutrophil gelatinase-associated lipocalin (uNGAL) and urine output at different time points

	Urine output 24 h prior to discontinuation	Urine output 6 h	Urine output 12 h	Urine output 24 h after discontinua-tion	uNGAL at discontinuation	uNGAL 6 h after discontinuation
	(OUC24pre)	(OUA6)	(OUA12)	(OUC24post)	uNGAL 0 h	uNGAL 6 h
AUC	0.86	0.89	0.92	0.84	0.80	0.81
Sensitivity	0.91	0.67	0.72	0.76	0.86	0.86
Specicificity	0.77	0.91	1.00	0.86	0.68	0.73
NPV	0.85	0.67	0.73	0.76	0.79	0.80
PPV	0.85	0.91	1.00	0.86	0.77	0.81
Cut-off, You-den’s index	210 ml	18 ml	24 ml	1260 ml	2403 μg/L	1650 μg/L

### Performance of single variable diagnostic tests

Of the single variable diagnostic tests described in Table 3, the best test using uNGAL was uNGAL 6 h having slightly better AUC, specificity and sensitivity (0.862 vs. 0.857) and also slightly better NPV. The best diagnostic test overall according to Youden’s index was OUA 12 (Youden’s index = 0.724) followed by OUC24pre (Youden’s index = 0.679). The latter also demonstrated by far the best sensitivity and NPV. When exploring the diagnostic performance of all permutations of 2-variable tests combining a uNGAL test with one of the tests for urine output using “or” and “and” operators (16 combinations), the highest Youden’s index and the highest sensitivity was seen for the combinations of uNGAL 6 h with UOC24pre.

### Performance of diagnostic tests combining uNGAL 6 h with UOC24pre

The “or” combination of uNGAL at 6 h and UOC24pre yielded the best sensitivity (0.97) and NPV (0.93). The 2-variable test combinations of uNGAL 6 h and UOC24pre using the cut-off values based on Youdens’s index (see Table 3) are presented in Table 4. The NRI (increment in Youden’s index) for the uNGAL 6 h and OUC24pre 2-variable models in Table 4 as compared to either of the single variable models of uNGAL 6 h and UOC24pre were:

- uNGAL 6 h *or* OUC24pre vs. uNGAL 6 h (0.966 + 0.591) - (0.862 + 0.727) = − 0.033
- uNGAL 6 h *and* OUC24pre vs uNGAL 6 h (0.793 + 0.909) - (0.862 + 0.727) = 0.113
- uNGAL 6 h *or* OUC24pre vs. UOC24pre (0.966 + 0.591) - (0.906 + 0.733) = − 0.123
- uNGAL 6 h *and* OUC24pre vs OUC24pre (0.793 + 0.909) - (0.906 + 0.733) = 0.023

**Table 4:** Test parameters and results of diagnostics tests combining the two variables, urine neutrophil gelatinase-associated lipocalin at 6 h (uNGAL 6 h) and urine output 24 h before CRRT discontinuation (OUC24pre)

Test	uNGAL> 1650 μg/L *or* urine output < 210 ml		uNGAL> 1650 μg/L *and* urine output < 210 ml	
	Non-recovery	Recovery	Non-recovery	Recovery
Test positive	28	9	23	2
Test negative	1	13	6	20
Sensitivity	0.97		0.79	
Specificity	0.59		0.91	
NPV	0.93		0.77	
PPV	0.76		0.92	

*Each of the 2 tests are to be seen as a complete diagnostic test with 2 variables.* E.g.*, for “uNGAL > 1650 um/L or urine output < 210 ml” the test is positive, if either or both “uNGAL > 1650” or “urine output < 210 ml” are true. The test is negative if neither of “uNGAL > 1650” or “urine output < 210 ml” are true. PPV = positive predictive value; NPV = negative predictive value*

Thus, only the “and” combination of the 2-variable model showed increased overall diagnostic performance when compared to the single parameter models. However, the “or” combination demonstrated the highest sensitivity and this test combination is therefore the best predictor for successful CRRT discontinuation.

A ROC curve comparing uNGAL 6-h and UOP24pre is available in the appendix.

## Discussion

In this prospective study, it was shown that for ICU patients with complex causes of AKI, uNGAL in combination with urine output was a better predictor of renal function recovery than either applied as a single variable test. Relative to the cessation of CRRT, the best prediction (NPV) of successful CRRT discontinuation was 93%, and was obtained with uNGAL 6 h combined with OUC24pre. Similarly, the best prediction (PPV) of continued need of dialysis was 92% and was obtained using the same two variables, but in another combination (Table 4).

The main implication of this is that, although uNGAL as a single variable was not shown to be diagnostically superior to urine output, it did add to the predictive ability of the latter. As such, this study may be taken as a proof of concept that uNGAL is a significant paraclinical parameter with regard to the evaluation of renal recovery and successful CRRT discontinuation in ICU patients.

Technically, the combination of uNGAL 6 h and UOC24pre (using the “and” operator) was a superior diagnostic test (increase in NRI) compared to either of the single variable models, uNGAL 6 h and UOC24pre. The test combination of uNGAL 6 h and UOC24 pre (using the “or” operator), however, had superior sensitivity and was therefore the better diagnostic test to predict successful CRRT discontinuation, although it was inferior (decrease in NRI) to the single variable tests.

Being able to predict the state of non-recovery in ICU dialysis patients as early as 6 h after CRRT discontinuation has several potential benefits. In addition to the benefit of reducing the time a given patient is without obligatory dialysis, there may also be an economic benefit by avoiding unnecessarily replacement of the dialysis filter.

We arbitrarily chose a “positive” test outcome to associate with continued renal failure (and dialysis dependency). Consequently, a “negative” diagnostic test using uNGAL and/or urine output (Tables 3 and 4) is a predictor of renal recovery (successful CRRT discontinuation).

The procedure in the ICU from which the patients in this study were recruited is that only patients who are ready to be discharged may be transferred from CRRT to HD. It follows that to initiate haemodialysis, the patient must have infection/inflammatory control and no longer require vasopressor or respiratory support. In this situation, the laboratory parameters indicating inflammation, including uNGAL, are expected to be low. To elaborate, patients eligible to initiate HD are past or almost past the phase of inflammation and acute kidney damage, i.e., the uNGAL level is declining or low, but the kidney function as assessed by serum creatinine, creatinine clearance and urine output is still not satisfactory.

If, in theory, it was possible to identify patients in need of haemodialysis at a point before CRRT cessation, the NPV of the combined uNGAL 6 h/OUC24pre test in our setting would be 100%, as 1 out of 14 patients with a negative test became permanently haemodialysis dependent (Table 4).

To date, only a very few studies have examined the ability of uNGAL to predict renal function recovery after dialysis in critically ill patients. In the study by Yang et al. [14], 102 patients were enrolled, and 8 biomarkers, including uNGAL, were analysed at 24 h after the discontinuation of dialysis. At 60 days after discontinuation, renal recovery was defined as a serum creatinine level < 0.5 mg/dl (84.2 μmol/L). The ability of uNGAL to predict renal recovery at this time point was poor, with an AUC of 50% [14]. However, Yang et al.’s [14] main purpose for measuring uNGAL was not, as in this study, the determination of the immediate need to restart dialysis but prognosticate lasting damage to the kidney. Another study [Biological Markers of Recovery for the Kidney (BioMark)] by Srisawat et al. [15] also indicated the ability of uNGAL to predict more lasting kidney damage. Finally, a study from 2019 by Stads et al. [16] included 92 patients and tested 7 variables, including uNGAL 2 days after the discontinuation of dialysis. Renal recovery was defined as freedom from dialysis 7 days after discontinuation. Urine NGAL was not significantly associated with renal recovery. However, it should be considered whether this study has clinical relevance. It is likely that, at 2 days after discontinuation, the physician already had a very good inclination of the need for dialysis.

There are several limitations of our study. The major limitation was the small number of included patients. This has 2 implications. With a limited number of patients in the different subgroups of recovery and non-recovery, the cut-off limits for uNGAL 6 h and UOC24pre become less certain, since the range of values of either inevitably will contain gaps rather than representing an unbroken continuum. With regard to the cut-off values representing the best NPV of the combined parameters, uNGAL 6 h at 1650 μg/L and UOC24pre at 210 mL were flanked by values of 1327 μg/L and 1863 μg/L and by 195 and 228 mL, respectively. The true cut-off values for the two parameters may therefore in reality lie close to one of their flanking values at either end. The other uncertainty arising from the limited number of patients is the confidence in the calculated NPV and PPV values given in Table 4. For example, the NPV at 93% is based on the ratio of 13/14 patients and will thus – based on the binomial distribution – have a 90% one-sided lower confidence limit at 75%. In conclusion, a larger study with more included patients is needed to confirm the findings of this study. Excluding or reclassifying (from “recovery” to “non-recovery”) the patient who was placed back on CRRT after 92 h did not significantly impact the uNGAL 6 h/ UOC24pre prediction model.

Secondly, a number of urine samples were missed for a multitude of reasons. This was mitigated by extrapolating 14 out of 51 values for uNGAL 6 h from either uNGAL 0 h or uNGAL 12 h using linear regression. Despite solid statistically significant correlations between log uNGAL 6 h values with those at 0 h and at 12 h, this is a source of uncertainty with regard to the true numeric value of the interpolated uNGAL 6 h values. However, the uNGAL 0 h and uNGAL 12 h values from which the uNGAL 6 h values were interpolated, yields the exact same test results in relation to uNGAL 0 h and uNGAL 12 h cut-off values, respectively, as is the case for the interpolated uNGAL 6 h values in relation to the uNGAL 6 h cut-off value. Therefore, interpolation did not change the conclusion of diagnostic test based on uNGAL 6 h, but it did allow for the “alignment” of all 51 uNGAL results to the 6 h time point.

The main strength of the study is that it focused on the main clinical issue, i.e., the turning point at which the physician acts and discontinues CRRT. How soon can the physician obtain a reliable estimate of whether the patient has regained renal function or whether dialysis has to be reinitiated? The study showed that the use of two variables, uNGAL and urine output, had greater clinical usefulness than the use of either variable as a single test. The generalizability of the study results appears to be quite good, especially because the included AKI patients were selected broadly. However, the results should be used with care in ICUs in which furosemide is given routinely while the patients are on dialysis.

## Conclusions

In the setting of a multidisciplinary ICU with patients who have complex causes underlying acute kidney failure, a predictive value of 93% for successful CRRT discontinuation was obtained with a combination of uNGAL at 6 h and cumulated urine output for 24 h prior to discontinuation, with cut-offs of 1650 μg/L and 210 mL, respectively.

## Supplementary information

**Additional file 1.** Appendix

## Data Availability

The datasets used and/or analysed during the current study are available from the corresponding author on reasonable request.

## Abbreviations

REC: Renal recovery
NREC-A: Non-recovery, all
NREC-H: Non-recovery, haemodialysis
NREC-C: Non-recovery; CRRT
UOC24pre: Cumulated urine output for 24 h prior to CRRT cessation
UOC24post: Cumulated urine output for 24 h after CRRT discontinuation
UOA6: Average hourly urine output the first 6 h after discontinuation
UOA12: Average hourly urine output the first 12 h after cessation
