# Supplementary material for: Urine neutrophil gelatinase-associated lipocalin and urine output as predictors of the successful discontinuation of continuous renal replacement therapy in critically ill patients with acute kidney injury
Source: BMC Nephrol. 2020 Aug 28;21:375. doi: 10.1186/s12882-020-02035-w (PMC7456074; doi:10.1186/s12882-020-02035-w)

**Appendix**

**Table 1.1** Recovery versus Non-recovery (Haemodialysis initiation)

| Clinical variables | Recovery  (n=22) | ~~Non-recovery; CRRT re-initiation~~  ~~(n=20)~~ | Non-recovery; Haemodialysis initiation (n=12) | P-value |
| --- | --- | --- | --- | --- |
| Male/female | 16/6 (73%/27%) | ~~11/9 (55%/45%)~~ | 9/3 (75%/25%) | 0.89 |
| Age, years | 77 [42-83] | ~~70 [51-81]~~ | 68 [30-83] | 0.65 |
| BMI, kg/m^2^ | 26 [19-35] | ~~27 [19-38]~~ | 25 [24-37] | 0.22 |
| SOFA, highest value | 13 [7-18] | ~~15 [11-19]~~ | 13 [8-21] | 0.98 |
| APACHE II | 27 [18-40] | ~~29 [21-43]~~ | 26 [18-38] | 0.68 |
| SAPS II | 56 [30-75] | ~~65 [44-86]~~ | 58 [38-78] | 0.63 |
| Hypertension | 9 (41%) | ~~11 (55%)~~ | 7 (58%) | 0.19 |
| Malignity | 6 (27%) | ~~10 (50%)~~ | 2 (17%) | 0.49 |
| CVVH/CVVHD | 0/22 (0/100%) | ~~4/16 (20/80%)~~ | 0/12 (0/100%) | — |
| RIFLE-F | 21 (95%)^a^ | ~~20 (100%)~~ | 12 (100%) | 0.45 |
| Furosemide, mg/day | 0 [0-554] | ~~80 [0-534]~~ | 100 [0-480] | 0.34 |
| Mean arterial pressure, mmHg | 69 [62-85] | ~~70 [63-93]~~ | 78 [59-113] | 0.16 |
| Creatinine, µmol/L | 186 [84-621] | ~~194 [87-531]~~ | 238 [90-765] | 0.55 |
| Creatinine clearance, ml/min | 19 [7-45] | ~~15 [8-27]~~ | 17 [5-26] | 0.10 |
| C-Reactive Protein (CRP), mg/L | 216 [7-454] | ~~139 [13-266]~~ | 108 [34-423] | 0.39 |
| Urine output, ml/hour ^b^ | 18 [0-50] | ~~8 [0-97]~~ | 0 [0-52] | 0.07 |
| Urine output, ml/day ^c^ | 398 [103-1488] | ~~326 [8-1750]~~ | 120 [13-2550] | 0.83 |
| Noradrenaline, µg/kg/min | 0.35 [0.09-1.26] | ~~0.28 [0.07-1.32]~~ | 0.12 [0.02-1.10] | 0.13 |
| Nephrotoxic medicine including contrast. | 8 (36%) | ~~12 (60%)~~ | 6 (50%) | 0.44 |
| Presumed primary cause of AKI:  Prerenal  Sepsis  Glomerulonephritis  Rhabdomyolysis  Microthrombosis/  vasculitis | 8 (36%)  12 (55%)  0  1 (4.5%)  1 (4.5%) | ~~5 (25%)~~  ~~13 (65%)~~  ~~0~~  ~~1 (5%)~~  ~~1 (5%)~~ | 2 (17%)  4 (33%)  1 (8%)  3 (25%)  2 (17%) | 0.11 |
| Primary reason for ICU admission:  Septic shock/sepsis  Trauma  AKI  Respiratory failure  Low cardiac output | 9 (41%)  1 (5%)  5 (23%)  4 (18%)  3 (14%) | ~~103(65%)~~  ~~2 (10%)~~  ~~1 (5%)~~  ~~2 (10%)~~  ~~2 (10%)~~ | 6 (51%)  0  2 (17%)  3 (34%)  1 (8%) | 0.80 |
| Mechanical ventilation, days | 4 [0-23] | ~~14[0-32]~~ | 2 [0-18] | 0.47 |
| ICU, days | 10 [2-32] | ~~17 [5-38]~~ | 9 [3-20] | 0.30 |
| ICU admission  to CRRT initiation, days | 1 [0-7.4] | ~~2 [0-5.9]~~ | 1 [0-12.1] | 0.41 |
| Urine-NGAL, µg/L | 2645 [279-27543] | ~~2894 [749-18537]~~ | 3619 [128-30170] | 0.76 |

**Table 2.1** Recovery versus Non-recovery (Haemodialysis initiation)

| Variable | n | Recovery  (n=22) | ~~Non-recovery;~~  ~~CRRT re-initiation~~  ~~(n=20)~~ | Non-recovery;  Haemodialysis initiation  (n=12) | p-value |
| --- | --- | --- | --- | --- | --- |
| Urine NGAL  time 0, µg/L  6 hrs, µg/L  12 hrs, µg/L  24 hrs, µg/L | 42  37  39  31 | 1370 [154-8002]  465 [56-3978]  436 [95-3780]  455 [100-3337] | ~~8934 [2095-40000]~~  ~~2966 [1985-29011]~~  ~~1976 [798-16972]~~  ~~2290 [874-17665]~~ | 8726 [360-35449]  2854 [120-6055]  2148 [54-3825]  295 [32-1624] | 0.08  0.20  0.14  0.42 |
| MAP  time 0, mmHg  6 hrs, mmHg  12 hrs, mmHg  24 hrs, mmHg | 54  52  50  46 | 80 [67-103]  78 [66-105]  75 [67-106]  75 [67-106] | ~~71 [62-94]~~  ~~73 [66-105]~~  ~~73 [65-105]~~  ~~75 [67-103]~~ | 82 [67-96]  78 [64-106]  80 [68-103]  83 [68-113] | 0.63  0.78  0.90  0.83 |
| Furosemide  6 hrs., mg  12 hrs., mg  24 hrs., mg | 52  50  46 | 70 [0-240]  95 [0-480]  150 [0-853] | ~~70 [0-246]~~  ~~95 [0-480]~~  ~~240 [15-936]~~ | 5 [0-240]  65 [0-480]  70 [0-936] | 0.90  0.69  0.68 |
| Use of other diuretics |  | 4 (18%) | ~~4 (20%)~~ | 6 (50%) | 0.14 |
| Urine output 24 hrs prior to CRRT (time 0) discontinuation, ml | 54 | 500 [87-2140] | ~~100 [31-533]~~ | 20 [0-575] | 0.0004 |
| Urine output after  discontinuation  6 hrs, ml/hr  12 hrs, ml/hr  24 hrs, ml | 52  50  46 | 65 [13-266]  85 [27-197]  2340 [828-4488] | ~~8 [0-47]~~  ~~10 [0-60]~~  ~~480 [0-2952]~~ | 3 [0-50]  3 [0-54]  240 [0-3828] | 0.006  <0.0001  0.003 |
| Time to re-initiation of CRRT, hrs |  |  | ~~24 [6-64]~~ |  |  |
| CRRT, days before discontinuation | 54 | 4 [2-10] | ~~8 [4-20]~~ | 5 [2-18] | 0.41 |
| Creatinine  At discontinuation, µmol/L  24 hrs after  discontinuation, µmol/L | 54  46 | 98 [51-250]  134 [67-340] | ~~130 [67-289]~~  ~~191 [119-409]~~ | 157 [82-314]  235 [135-433] | 0.18  0.04 |
| Creatinine clearance  At discontinuation, ml/min 24 hrs after  discontinuation, ml/min | 54  46 | 62 [17-90]  38 [16-82] | ~~43 [21-85]~~  ~~28 [15-52]~~ | 37 [18-75]  22 [12-41] | 0.05  0.004 |
| C-reactive protein (CRP)  At discontinuation, mg/L 24 hrs after  discontinuation, mg/L | 54  46 | 135 [35-267]  125 [26-238] | ~~89 [22-260]~~  ~~100 [20-320]~~ | 80 [16-172]  65 [13-152] | 0.03  0.04 |

**Table 1.2** Recovery versus Non-recovery (CRRT reinitiation)

| Clinical variables | Recovery  (n=22) | Non-recovery; CRRT re-initiation  (n=20) | ~~Non-recovery; Haemodialysis initiation (n=12)~~ | P-value |
| --- | --- | --- | --- | --- |
| Male/female | 16/6 (73%/27%) | 11/9 (55%/45%) | ~~9/3 (75%/25%)~~ | 0.23 |
| Age, years | 77 [42-83] | 70 [51-81] | ~~68 [30-83]~~ | 0.95 |
| BMI, kg/m^2^ | 26 [19-35] | 27 [19-38] | ~~25 [24-37]~~ | 0.43 |
| SOFA, highest value | 13 [7-18] | 15 [11-19] | ~~13 [8-21]~~ | 0.80 |
| APACHE II | 27 [18-40] | 29 [21-43] | ~~26 [18-38]~~ | 0.39 |
| SAPS II | 56 [30-75] | 65 [44-86] | ~~58 [38-78]~~ | 0.04 |
| Hypertension | 9 (41%) | 11 (55%) | ~~7 (58%)~~ | 0.48 |
| Malignity | 6 (27%) | 10 (50%) | ~~2 (17%)~~ | 0.14 |
| CVVH/CVVHD | 0/22 (0/100%) | 4/16 (20/80%) | ~~0/12 (0/100%)~~ | 0.03 |
| RIFLE-F | 21 (95%)^a^ | 20 (100%) | ~~12 (100%)~~ | 0.33 |
| Furosemide, mg/day | 0 [0-554] | 80 [0-534] | ~~100 [0-480]~~ | 0.44 |
| Mean arterial pressure, mmHg | 69 [62-85] | 70 [63-93] | ~~78 [59-113]~~ | 0.80 |
| Creatinine, µmol/L | 186 [84-621] | 194 [87-531] | ~~238 [90-765]~~ | 0.61 |
| Creatinine clearance, ml/min | 19 [7-45] | 15 [8-27] | ~~17 [5-26]~~ | 0.67 |
| C-Reactive Protein (CRP), mg/L | 216 [7-454] | 139 [13-266] | ~~108 [34-423]~~ | 0.14 |
| Urine output, ml/hour ^b^ | 18 [0-50] | 8 [0-97] | ~~0 [0-52]~~ | 0.59 |
| Urine output, ml/day ^c^ | 398 [103-1488] | 326 [8-1750] | ~~120 [13-2550]~~ | 0.75 |
| Noradrenaline, µg/kg/min | 0.35 [0.09-1.26] | 0.28 [0.07-1.32] | ~~0.12 [0.02-1.10]~~ | 0.81 |
| Nephrotoxic medicine including contrast. | 8 (36%) | 12 (60%) | ~~6 (50%)~~ | 0.13 |
| Presumed primary cause of AKI:  Prerenal  Sepsis  Glomerulonephritis  Rhabdomyolysis  Microthrombosis/  vasculitis | 8 (36%)  12 (55%)  0  1 (4,5%)  1 (4,5%) | 5 (25%)  13 (65%)  0  1 (5%)  1 (5%) | ~~2 (17%)~~  ~~4 (33%)~~  ~~1 (8%)~~  ~~3 (25%)~~  ~~2 (17%)~~ | 0.89 |
| Primary reason for ICU admission:  Septic shock/sepsis  Trauma  AKI  Respiratory failure  Low cardiac output | 9 (41%)  1 (5%)  5 (23%)  4 (18%)  3 (14%) | 103(65%)  2 (10%)  1 (5%)  2 (10%)  2 (10%) | ~~6 (51%)~~  ~~0~~  ~~2 (17%)~~  ~~3 (34%)~~  ~~1 (8%)~~ | 0.38 |
| Mechanical ventilation, days | 4 [0-23] | 14[0-32] | 2 [0-18] | 0.05 |
| ICU, days | 10 [2-32] | 17 [5-38] | 9 [3-20] | 0.05 |
| ICU admission  to CRRT initiation, days | 1 [0-7.4] | 2 [0-5.9] | 1 [0-12.1] | 0.52 |
| Urine-NGAL, µg/L | 2645 [279-27543] | 2894 [749-18537] | 3619 [128-30170] | 0.51 |

**Table 2.2** Recovery versus Non-recovery (CRRT re-initiation).

| Variable | n | Recovery  (n=22) | Non-recovery;  CRRT re-initiation  (n=20) | ~~Non-recovery;~~  ~~Haemodialysis initiation~~  ~~(n=12)~~ | p-value |
| --- | --- | --- | --- | --- | --- |
| Urine NGAL  time 0, µg/L  6 hrs, µg/L  12 hrs, µg/L  24 hrs, µg/L | 42  37  39  31 | 1370 [154-8002]  465 [56-3978]  436 [95-3780]  455 [100-3337] | 8934 [2095-40000]  2966 [1985-29011]  1976 [798-16972]  2290 [874-17665] | ~~8726 [360-35449]~~  ~~2854 [120-6055]~~  ~~2148 [54-3825]~~  ~~295 [32-1624]~~ | 0.0001  <0.0001  0.0001  0.14 |
| MAP  time 0, mmHg  6 hrs, mmHg  12 hrs, mmHg  24 hrs, mmHg | 54  52  50  46 | 80 [67-103]  78 [66-105]  75 [67-106]  75 [67-106] | 71 [62-94]  73 [66-105]  73 [65-105]  75 [67-103] | ~~82 [67-96]~~  ~~78 [64-106]~~  ~~80 [68-103]~~  ~~83 [68-113]~~ | 0.11  0.21  0.30  0.41 |
| Furosemide  6 hrs., mg  12 hrs., mg  24 hrs., mg | 52  50  46 | 70 [0-240]  95 [0-480]  150 [0-853] | 70 [0-246]  95 [0-480]  240 [15-936] | ~~5 [0-240]~~  ~~65 [0-480]~~  ~~70 [0-936]~~ | 0.39  0.61  0.17 |
| Use of other diuretics |  | 4 (18%) | 4 (20%) | ~~6 (50%)~~ | 0.88 |
| Urine output 24 hrs prior to CRRT (time 0) discontinuation, ml | 54 | 500 [87-2140] | 100 [31-533] | ~~20 [0-575]~~ | 0.006 |
| Urine output after  discontinuation  6 hrs, ml/hr  12 hrs, ml/hr  24 hrs, ml | 52  50  46 | 65 [13-266]  85 [27-197]  2340 [828-4488] | 8 [0-47]  10 [0-60]  480 [0-2952] | ~~3 [0-50]~~  ~~3 [0-54]~~  ~~240 [0-3828]~~ | 0.0007  <0.0001  0.001 |
| Time to re-initiation of CRRT, hrs |  |  | 24 [6-64] |  |  |
| CRRT, days before discontinuation | 54 | 4 [2-10] | 8 [4-20] | ~~5 [2-18]~~ | 0.52 |
| Creatinine  At discontinuation, µmol/L  24 hrs after  discontinuation, µmol/L | 54  46 | 98 [51-250]  134 [67-340] | 130 [67-289]  191 [119-409] | ~~157 [82-314]~~  ~~235 [135-433]~~ | 0.68  0.27 |
| Creatinine clearance  At discontinuation, ml/min 24 hrs after  discontinuation, ml/min | 54  46 | 62 [17-90]  38 [16-82] | 43 [21-85]  28 [15-52] | ~~37 [18-75]~~  ~~22 [12-41]~~ | 0.18  0.06 |
| C-reactive protein (CRP)  At discontinuation, mg/L 24 hrs after  discontinuation, mg/L | 54  46 | 135 [35-267]  125 [26-238] | 89 [22-260]  100 [20-320] | ~~80 [16-172]~~  ~~65 [13-152]~~ | 0.34  0.99 |


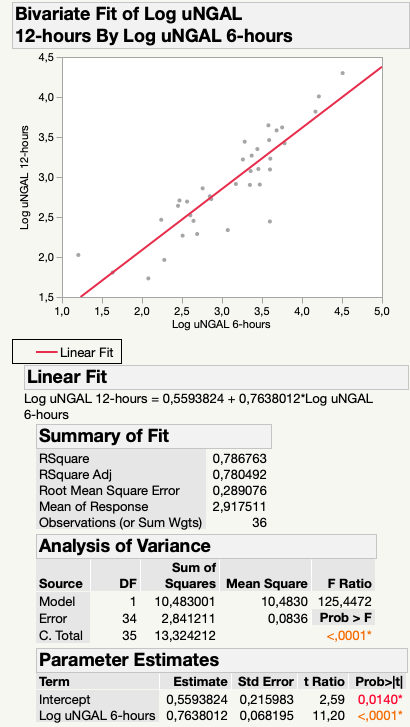


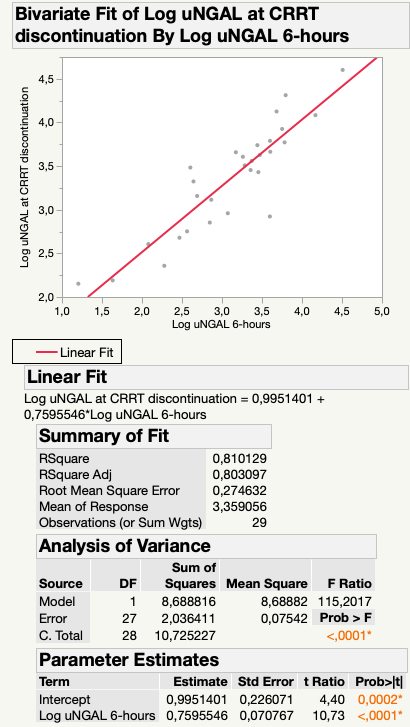


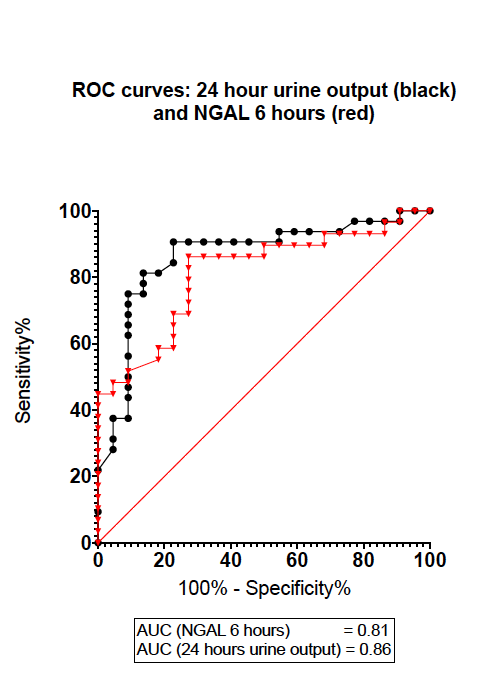

Supplement: Supplementary file 1 — Additional file 1. Appendix [file 12882_2020_2035_MOESM1_ESM.docx]
